# Pulmonary oxygen uptake and muscle deoxygenation kinetics during heavy intensity cycling exercise in patients with emphysema and idiopathic pulmonary fibrosis

**DOI:** 10.1186/s12890-017-0364-z

**Published:** 2017-01-31

**Authors:** Melitta A. McNarry, Nicholas K. Harrison, Tom Withers, Narendra Chinnappa, Michael J. Lewis

**Affiliations:** 1 A-STEM, College of Engineering, Swansea University, Swansea, UK; 2 College of Medicine, Swansea University, Swansea, UK; 3 Respiratory Unit, Morriston Hospital, Swansea, UK

**Keywords:** Respiratory disease, $\overset{.}{V}$ O_2_ kinetics, NIRS, Cycle

## Abstract

**Background:**

Little is known about the mechanistic basis for the exercise intolerance characteristic of patients with respiratory disease; a lack of clearly defined, distinct patient groups limits interpretation of many studies. The purpose of this pilot study was to investigate the pulmonary oxygen uptake ($\overset{.}{V}$ O_2_) response, and its potential determinants, in patients with emphysema and idiopathic pulmonary fibrosis (IPF).

**Methods:**

Following a ramp incremental test for the determination of peak $\overset{.}{V}$ O_2_ and the gas exchange threshold, six emphysema (66 ± 7 years; FEV_1,_ 36 ± 16%), five IPF (65 ± 12 years; FEV_1_, 82 ± 11%) and ten healthy control participants (63 ± 6 years) completed three repeat, heavy-intensity exercise transitions on a cycle ergometer. Throughout each transition, pulmonary gas exchange, heart rate and muscle deoxygenation ([HHb], patients only) were assessed continuously and subsequently modelled using a mono-exponential with ($\overset{.}{V}$ O_2_, [HHb]) or without (HR) a time delay.

**Results:**

The $\overset{.}{V}$ O_2_ phase II time-constant (τ) did not differ between IPF and emphysema, with both groups significantly slower than healthy controls (Emphysema, 65 ± 11; IPF, 69 ± 7; Control, 31 ± 7 s; *P* < 0.05). The HR τ was slower in emphysema relative to IPF, with both groups significantly slower than controls (Emphysema, 87 ± 19; IPF, 119 ± 20; Control, 58 ± 11 s; *P* < 0.05). In contrast, neither the [HHb] τ nor [HHb]:O_2_ ratio differed between patient groups.

**Conclusions:**

The slower $\overset{.}{V}$ O_2_ kinetics in emphysema and IPF may reflect poorer matching of O_2_ delivery-to-utilisation. Our findings extend our understanding of the exercise dysfunction in patients with respiratory disease and may help to inform the development of appropriately targeted rehabilitation strategies.

## Background

Pulmonary oxygen uptake ($\overset{.}{V}$ O_2_) kinetics provides an insight into the integrated capacity of an organism to transport and utilize oxygen to support an increased rate of energy turnover in contracting myocytes [1]. This dynamic $\overset{.}{V}$ O_2_ response is highly sensitive to both advantageous and deleterious adaptations. For example, the $\overset{.}{V}$ O_2_ kinetics of patients with chronic obstructive pulmonary disease (COPD) are slower than their age-matched controls [e.g. 2–4] but this can be ameliorated, to some extent, by exercise training [3, 5]. This slow $\overset{.}{V}$ O_2_ kinetic response has been suggested to be mechanistically associated with the reduced exercise tolerance typical of this population [6–10].

The pathological basis for the slower dynamic $\overset{.}{V}$ O_2_ response in COPD remains uncertain, with little consensus within the literature regarding the principal determinants of the slower $\overset{.}{V}$ O_2_ kinetics observed during moderate intensity exercise. Skeletal muscle dysfunction characterized by a decreased capillary network [11], reduced myoglobin level [12], abnormal oxidative capacity [13, 14] and altered muscle fibre type distribution (increased relative proportion of type II, glycolytic muscle fibres [7]) have been suggested to be associated with the slower $\overset{.}{V}$ O_2_ kinetics [2, 15] and positive responses to training reported in some previous studies [5, 8]. However, others have suggested that the cardiovascular derangements often present in those with COPD may engender an oxygen delivery limitation leading to the slower dynamic $\overset{.}{V}$ O_2_ response observed [3, 16], a contention supported by the limited evidence available regarding the $\overset{.}{V}$ O_2_ response during heavy intensity exercise [4]. Further interpretation of these findings is limited, however, by methodological limitations such as the reliance on a single exercise bout for the derivation of $\overset{.}{V}$ O_2_ kinetics, which may not reflect the true response [17], and significant potential variability in patient populations. Specifically, COPD is an umbrella term that encompasses different pathological entities including chronic bronchitis, emphysema, small airways disease and respiratory bronchiolitis interstitial lung disease [18]. Some or all of these entities may be present within an individual in varying proportion.

In contrast to emphysema, there is a paucity of data on exercise response of patients with fibrotic lung disease [19]. Idiopathic pulmonary fibrosis (IPF) is a common life-limiting condition characterized by progressive deposition of extracellular matrix in the alveolar interstitium. This condition is thought to arise from aberrant wound healing following alveolar epithelial injury [20]. IPF results in symptoms of increasing breathlessness, functional decline and eventual death, with a median survival time of approximately three years following diagnosis [21].

While emphysema and IPF are distinct pathological conditions, both result in a loss of functional alveolar units and impaired gas exchange. However, the destructive process of emphysema causes a loss of elastic recoil in the lungs, resulting in gas trapping and airflow obstruction on expiration with a relative fall in forced expiratory volume (FEV_1_) compared to forced vital capacity (FVC). In contrast, increased extracellular matrix in the alveolar walls of patients with IPF causes the lungs to fibrose with a consequent reduction in lung volumes and a matched fall in FEV_1_ and FVC. Therefore, the vital capacity of patients with IPF becomes progressively decreased without affecting airflow [20]. The differential manifestation of these pathological adaptations with regards to the $\overset{.}{V}$ O_2_ kinetics response is presently unknown, although it is perhaps pertinent to note the central role of arterial desaturation in exercise intolerance reported in patients with IPF [22, 23].

In this context, the purpose of the present pilot study was to investigate the $\overset{.}{V}$ O_2_ kinetics response, during heavy intensity exercise in well-characterized patients with emphysema and IPF.

## Methods

### Participants

Overall, eleven patients and ten healthy volunteers (62.8 ± 6.4 years; 9 males) who served as a control group completed this study (Table 1). The patient group comprised of six patients with emphysema (66.4 ± 7.4 years; 5 male; GOLD classification stage II, *n* = 1; stage III, *n* = 3 and stage IV, *n* = 2) and five patients with IPF (64.7 ± 11.6 years; 4 males). One patient with emphysema and two with IPF dropped out due to issues unrelated to the study. Furthermore, one patient with Emphysema was excluded from the subsequent analyses due to concern regarding the quality of the data and the values derived.

**Table 1:** Anthropometric characteristics and lung function according to participant condition

	Healthy	Emphysema	IPF
Age (yrs)	62.8 ± 6.4	66.4 ± 7.4	64.7 ± 11.6
Stature (m)	1.74 ± 0.11	1.67 ± 0.14	1.71 ± 0.07
Body Mass (kg)	76.3 ± 13.9	68.4 ± 8.9	79.0 ± 16.3
BMI (kg · m^−2^)	25.1 ± 3.3	24.6 ± 2.8	26.7 ± 3.5
BF (%)	26 ± 6	27 ± 9	28 ± 5
FEV_1_ (%)	-	51 ± 25	89 ± 11^a^
FVC (%)	-	94 ± 27	97 ± 24
FEV_1_/FVC	-	50 ± 14	97 ± 16^a^
DLco (%)	-	46 ± 17	47 ± 17
Kco (%)	-	55 ± 10	65 ± 25

Mean ± SD. *BMI*: body mass index; *BF*: body fat; *FEV1*: Forced Expiratory Volume in one second; *FVC*: Forced Vital Capacity; *DLco*: carbon monoxide transfer factor; *Kco*: carbon monoxide transfer coefficient. All parameters of lung function are expressed as mean percent predicted for age and height [64, 65]. ^a^Significant difference between Emphysema and IPF

None of the patients in either group were current smokers and none had clinical evidence of pulmonary hypertension. No patient had suffered from a respiratory infection or an acute exacerbation of their condition over the six months prior to the study. None of the patients had been prescribed oral corticosteroids or antibiotics during this time.

Inclusion criteria for patients with emphysema were: a history of cigarette smoking, FEV_1_/FVC ratio <0.7, and resting arterial PO_2_ > 60 Torr in room air. In addition, all emphysema patients had radiographic/CT evidence of hyperinflation and destruction of lung parenchyma typical of emphysema. All six patients were taking 18 μg of inhaled tiotropium daily and five patients were also taking an inhaled corticosteroid/long acting beta-2 agonist combination, comprising 500 μg of fluticasone and 50 μg of salmeterol twice daily.

All patients with IPF fulfilled the 2011 American Thoracic Society/European Respiratory Society criteria for this diagnosis. In particular, all IPF patients had clinical, radiological and physiological features consistent with a diagnosis of IPF. Each patient had undergone high resolution computed tomography (HRCT) to confirm the presence of pulmonary fibrosis, with predominant reticular or honeycomb pattern in the subpleural regions of the lung bases and little or no ground glass shadowing. One patient had undergone a surgical lung biopsy, which showed the histological pattern of usual interstitial pneumonia.

The control group comprised healthy volunteers who were non-smokers. All were physically active but none were involved in organized sports.

All participants in the study gave written, informed consent and the study protocol was approved by the local NHS Ethics Committee (Ref number: 102547), which adhered to the Declaration of Helsinki.

### Procedures

The participants were asked to arrive at the laboratory in a rested state, ~2 h postprandial and to avoid strenuous exercise in the 24 h preceding each testing session. The participants also refrained from taking caffeine for 6 h and alcohol for 24 h before each test, respectively. All the tests were performed at the same time of day (±2 h) and exercise testing was conducted using an electronically braked cycle ergometer (Lode Excalibur, Groningen, Netherlands).

### Incremental Test

The incremental exercise test began with 3 min cycling at 70 – 80 rpm against no additional resistance, followed by increments of 6–10 W · min–1 and 20–30 W · min^−1^ for patients and controls, respectively, until exhaustion. These ramp rates were chosen on the basis of each participant’s self-reported history of physical activity and symptoms and are in accord with those utilized in Chiappa et al. [4] in patients with COPD. The participants were asked to maintain a cadence of 70–80 rpm. Breath-by-breath pulmonary gas-exchange data were collected continuously during the incremental exercise tests (Jaeger, Oxycon Pro, Carefusion, San Diego). The highest average $\overset{.}{V}$ O_2_ measured over 10 s before exhaustion was taken as the peak $\overset{.}{V}$ O_2_ ($\overset{.}{V}$ O_2_ peak). The gas exchange threshold (GET) was determined as the $\overset{.}{V}$ O_2_ at which there was 1) a non-linear increase in carbon dioxide production (VCO_2_) relative to $\overset{.}{V}$ O_2_ and 2) an increase in minute ventilation (VE)/ $\overset{.}{V}$ O_2_ without an increase in VE/VCO_2_.

### Step Exercise Tests

For the determination of $\overset{.}{V}$ O_2_, heart rate (HR) and deoxyhemaglobin ([HHb]) kinetics, participants completed a series of “step” tests. The protocol, which was performed three times on separate days a minimum of 24 h apart, comprised 6 min of pedaling with no external resistance followed by an abrupt transition to the target heavy intensity work rate which was maintained for a further 6 mins. The heavy intensity work rate was calculated as the work rate that would elicit a $\overset{.}{V}$ O_2_ of 40% of the difference between the GET and peak $\overset{.}{V}$ O_2_ (∆40%). Following the observation of the low mean work rate associated with Δ40 % in this population (37 ± 12 w), no external resistance was added to the baseline period to prevent further reductions in the already small response amplitude. According to the manufacturer guidelines, “unloaded” pedaling is equivalent to 10w at 70 rpm. Throughout each exercise bout, participants maintained a cadence of 70–80 rpm.

### Measurements

During the exercise tests, the participants wore a facemask and breathed through an impeller turbine assembly (Jaeger Triple V, Hoechberg, Germany). The inspired and expired gas volumes and gas concentration signals were continuously sampled at 100 Hz. These analyzers were calibrated before each test with gases of known concentrations, and the turbine volume transducer was calibrated using a 3 l syringe (Hans Rudolph, Kansas City, MO). The volume and concentration signals were time-aligned by accounting for the delay in capillary gas transit and analyzer rise-time relative to the volume signal. HR was measured throughout the exercise tests using a three lead echocardiogram (ECG; Reynolds Lifecard CF digital Holter recorder, Spacelabs Healthcare Ltd., UK). The ECG leads were positioned in the modified V5, CC5, modified V5R electrode configuration. This system provided ECG data with a sample accuracy of 2.5 mV (magnitude of least significant bit; 12-bit resolution) and 1024 Hz sampling frequency.

Additionally, the oxygenation status of the right *m. vastus lateralis* was monitored during each transition using a commercially available near-infrared system (Portamon, Artinis Medical Systems, The Netherlands). This system consists of an emission probe which has three light sources and emits two wavelengths of light (760 and 850 nm) and a photon detector. The intensity of incident and transmitted light was recorded continuously at 10 Hz and used to estimate the concentration changes relative to zero-set baseline levels for oxygenated, deoxygenated, and total hemoglobin. The [HHb] was used as an indicator of oxygen extraction within the field of interrogation [e.g. 24, 25]. The contribution of myoglobin to the NIRS signal is currently unresolved and so the [HHb] signal described herein should be considered to refer to the combined concentration of both deoxygenated hemoglobin and myoglobin. Given recent evidence regarding the issues of proportionality between the $\overset{.}{V}$ O_2_ and [HHb] response to step exercise, the absolute amplitude changes were not considered [26]. The muscle was initially cleaned and the portable probe strapped to the skin at the midpoint of the muscle using physiotherapists’ tape (Kinesio Tex Gold). To ensure the device remained stationary during exercise, and to minimize the interference of extraneous light with the near-infrared signal, a bandage was wrapped around the leg to enclose the probe.

### $\overset{.}{V}$ O_2_ kinetics analysis

Initially, the breath-by-breath $\overset{.}{V}$ O_2_ responses to each step transition were visually examined to remove any errant breaths caused by coughing, swallowing, sighing etc., using a 5-s moving average to identify points lying in excess of four standard deviations from the local mean. Subsequently, each transition was interpolated to 1-s intervals, time aligned to the start of exercise and averaged to produce a single response profile. Following baseline correction, a mono-exponential model with a time delay (Eq. 1) was then applied to this averaged response:

1 $$ \overset{.}{V}{O}_{2(t)}={A}_1\cdot \left(1-{e}^{-\left(t-\delta \right)/{\tau}_1}\right) $$

where $\overset{.}{V}$ O_2_ is the increase in $\overset{.}{V}$ O_2_ at time t above the baseline value (calculated as the mean $\overset{.}{V}$ O_2_ from the first 45 s of the last minute of baseline pedaling), and A_1_, δ_1_ and τ_1_ are the primary component amplitude, time delay (which was allowed to vary freely), and time constant, respectively. Kinetic variables (A_1_, δ_1_ and τ_1_) and their 95% confidence intervals were determined by least squares non-linear regression analysis (Graphpad Prism, Graphpad Software, San Diego, CA). In accord with previous studies [27–29], a mono-exponential model was used because a bi-exponential model $\left(\varDelta \overset{.}{V}{O}_{2(t)}={A}_1\cdot \left(1-{e}^{-\left(t-{\delta}_1\right)/{\tau}_1}\right)+{A}_2\cdot \left(1-{e}^{-\left(t-{\delta}_2\right)/{\tau}_2}\right)\right)$ was found to produce an inferior and ambiguous fit. Indeed, given that the purpose of the bi-exponential model is, at least in part, to identify and characterize the slow component, this failure to accurately fit the data is likely attributable to the negligible slow component evident in our participants, as verified by methods previously described elsewhere [30]. Briefly, purpose-designed LabVIEW software was used which iteratively fitted a single exponential function to the $\overset{.}{V}$ O_2_ data until the window encompassed the entire exercise response. The resulting phase II time constants were plotted against time to identify the point at which the phase II time constant consistently deviated from the previously “flat” profile. The amplitude of the $\overset{.}{V}$ *O*_*2*_ slow component was subsequently determined by calculating the difference between the end exercise $\overset{.}{V}$ *O*_*2*_ and the sum of the primary amplitude and baseline $\overset{.}{V}$ O_2_. This was expressed both in absolute terms and relative to end-exercise $\overset{.}{V}$ O_2_. Finally, the mean response time (MRT), which reflects the time course of the entire $\overset{.}{V}$ O_2_ response, was calculated by fitting a single exponential curve with no time delay to all data from *t* = 0.

### [HHb] & HR Kinetics Analysis

The [HHb] and HR responses to exercise were also modelled. The responses to each transition were interpolated to 1 s intervals, time-aligned and averaged to produce a single data set. The time delay for the [HHb] response ([HHb] TD) was identified as the time after exercise onset at which [HHb] began a systematic increase above the nadir value. The [HHb] data were subsequently fitted with a single exponential with a time delay (Eq. 1); the fitting window started at the [HHb] TD and finished at the end of the exercise transition. In accord with previous studies [e.g. 29, 31–33], the HR response was modelled by a mono-exponential without a time delay (Eq. 2) with the fitting window started at *t* = 0.

2 $$ \varDelta H{R}_{(t)}={A}_1\cdot \left(1-{e}^{-\left(t/{\tau}_1\right)}\right) $$

where ∆HR is the increase in heart rate at time *t* above the baseline (calculated as the mean heart rate from the first 45 s of the last minute of baseline pedaling), and A_1_ and τ_1_ are the primary component amplitude and time constant, respectively. The [HHb] time delay and τ were summed, giving the effective [HHb] τ’ that reflects the overall time course of the [HHb] response from the onset of the step transition. The [HHb]/ $\overset{.}{V}$ O_2_ ratio was also calculated to provide a general index of the “excess” (relative to the steady-state values) O_2_ extraction for a given $\overset{.}{V}$ O_2_ [34]_._ Specifically, values >1.0 reflect a greater reliance on fractional O_2_ extraction compared with the exercise steady-state (values = 1.0) and reflects a poorer local O_2_ delivery relative to muscle O_2_ utilization in the area of interrogation. The second-by-second [HHb] and $\overset{.}{V}$ O_2_ data were normalised for each participant and the $\overset{.}{V}$ O_2_ left-shifted by 20s to account for the cardiodynamic phase, synchronizing the datasets for exercise onset. Subsequently, both data sets were averaged into 5 s time bins and the ratio of [HHb]: $\overset{.}{V}$ O_2_ was calculated for each time bin from 20 s to the end of exercise. The average of all the individual time bins was calculated to produce an overall “mean” ratio.

### Statistics

Between groups differences (healthy, emphysema or IPF) were assessed using an ANOVA with subsequent post-hoc comparisons using Tukey’s test where appropriate to identify the location of significant differences. Pearson’s product moment correlation coefficients were used to analyze the relationships between key variables. All statistical analyses were conducted using PASW Statistics 18 (SPSS, Chicago, IL). All data are presented as means ± SD. Statistical significance was accepted when *P* ≤ 0.05.

## Results

### Peak Exercise

Participant’s peak exercise values are shown in Table 2. Both patient groups demonstrated a significantly lower peak $\overset{.}{V}$ O_2_ than their healthy counterparts in absolute and relative terms. Emphysema patients had lower absolute peak $\overset{.}{V}$ O_2_ than IPF patients, but this difference was eliminated when expressed relative to fat free mass. The absolute GET was significantly higher in the healthy participants with no difference evident between emphysema and IPF patients. When the GET was expressed relative to peak $\overset{.}{V}$ O_2_, there were no differences between participant groups. There were no differences between emphysema and IPF patients in peak tidal volume (V_T_), breathing frequency or minute ventilation (V_E_). IPF demonstrated significantly lower values than healthy participants for both V_T_ (1.71 ± 0.63 vs. 3.08 ± 0.78 l∙min^−1^, respectively; *P* = 0.018) and V_E_ (59.2 ± 31.2 vs. 101.4 ± 29.2 l∙min^−1^, respectively; *P* = 0.042), whist V_E_ was significantly lower in emphysema than healthy participants (32.0 ± 13.7 vs. 101.4 ± 29.2 l∙min^−1^, respectively; *P* = 0.001).

**Table 2:** Peak exercise response in healthy participants and in patients with emphysema and IPF

	Healthy	Emphysema	IPF
Peak $\overset{.}{V}$_O2_ (l · min^−1^)	2.66 ± 0.70	1.35 ± 0.13^a^	1.41 ± 0.13^ab^
Peak $\overset{.}{V}$ O_2_ (l · kg^-0.66^ · min^−1^)	2.06 ± 0.62	1.04 ± .023^a^	1.06 ± 0.33^a^
GET (l · min^−1^)	1.48 ± 0.50	0.80 ± 0.11^a^	0.95 ± 0.15^a^
GET (%)	56 ± 12	57 ± 4	67 ± 7
Peak HR (b · min^−1^)	177 ± 12	142 ± 17^*^	142 ± 31^a^
Time to Exhaustion (min)	12.9 ± 3.2	9.5 ± 0.8	11.7 ± 3.5

Mean ± S.D. $\overset{.}{V}$ O_2_: oxygen uptake; *GET*: gas exchange threshold. ^a^Significant difference relative to healthy participants (*P* < 0.05) ^b^Significant difference between emphysema and IPF patients

### Constant work rate exercise

**Fig. 1:**
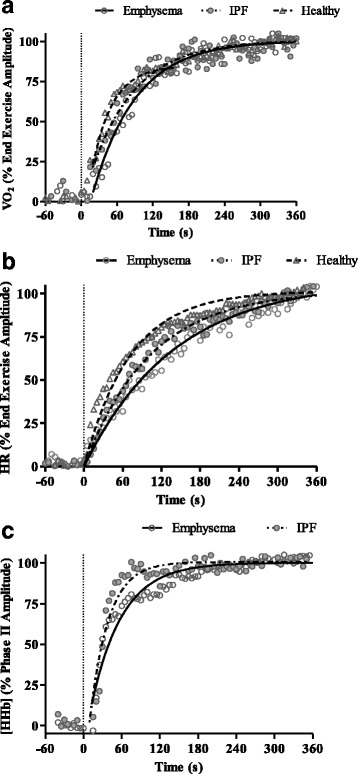
Pulmonary $\overset{.}{V}$ O_2_, HR and [HHb] responses during heavy intensity exercise. A) Pulmonary oxygen uptake response B) heart rate and C) deoxyheamaglobin responses during heavy intensity exercise in a representative patient with emphysema (open circles), IPF (closed circles) and a healthy participant (open triangles). The solid lines represent the mono-exponential model fit and dashed lines the onset of the increased work rate

**Fig. 2:**
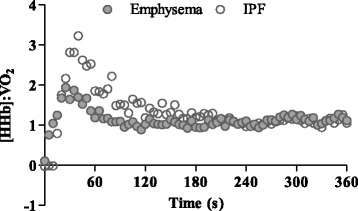
Representative profiles for the adjustment of [HHb]: $\overset{.}{V}$ O_2_ during the step transition to heavy intensity exercise

As summarized in Table 3 and illustrated in Fig. 1, the dynamic $\overset{.}{V}$ O_2_ response was significantly influenced by both lung diseases. Specifically, whilst emphysema and IPF patients did not differ from each other, both showed a significantly lower phase II amplitude and relative slow component amplitude and slower phase II τ and MRT compared to healthy participants. In contrast, in addition to being slower than healthy participants, emphysema patients evidenced a significantly slower HR response following the onset of exercise. Emphysema and IPF patients demonstrated a similar [HHb] τ and effective [HHb] τ’. The overall [HHb]: $\overset{.}{V}$ O_2_ ratio did not differ significantly between IPF and emphysema patients. Irrespective of patient group, there was a transient overshoot in the [HHb]: $\overset{.}{V}$ O_2_ response following the step change in exercise demand (Fig. 2).

**Table 3:** Pulmonary $\overset{.}{V}$ O_2_, HR and [HHb] responses during heavy intensity exercise according to participant condition

	Healthy	Emphysema	IPF
Baseline $\overset{.}{V}$ O_2_	0.62 ± 0.9	0.60 ± 0.12	0.75 ± 0.18
$\overset{.}{V}$ O_2_ Amp (l · min^−1^)	1.48 ± 0.54	0.40 ± 0.11^a^	0.41 ± 0.14^a^
$\overset{.}{V}$ O_2_ TD (s)	15 ± 5	11 ± 7	11 ± 7
$\overset{.}{V}$ O_2_ τ (s)	31 ± 7	65 ± 11^a^	69 ± 7^a^
$\overset{.}{V}$ O_2_ MRT (s)	52 ± 12	81 ± 18^a^	92 ± 19^a^
$\overset{.}{V}$ O_2_ SC Amp (l · min^−1^)	0.19 ± 0.10	0.01 ± 0.01^a^	0.01 ± 0.01^a^
$\overset{.}{V}$ O_2_ SC Amp (% EE $\overset{.}{V}$ O_2_)	7 ± 3	1 ± 1^a^	1 ± 1^a^
HR Amp (beats · min^−1^)	58 ± 11	22 ± 10^a^	21 ± 10^a^
HR τ (s)	36 ± 12	119 ± 20^a^	87 ± 19^ab^
[HHb] TD (s)	NA	18 ± 9	15 ± 2
[HHb] τ (s)	NA	51 ± 23	35 ± 29
[HHb] τ’ (s)	NA	69 ± 15	54 ± 35
[HHb]: $\overset{.}{V}$ O_2_ ratio	NA	1.07 ± 0.14	1.21 ± 0.07
Work rate (W)	155 ± 56	37 ± 12	64 ± 9

Mean ± S.D. (95% confidence intervals). $\overset{.}{V}$ O_2_, oxygen uptake; *Amp*: Amplitude; *TD*: time delay; *τ*: phase II time constant; *MRT*: mean response time; *SC*: slow component; *EE*: end exercise; *HR*: heart rate; *NA*: data not available

^a^Significant difference relative to healthy participants (*P* < 0.05); ^b^Significant difference between patients with Emphysema and IPF (*P* < 0.05)

There were no significant differences in steady state V_T_ (1.85 ± 0.56, 1.59 ± 0.48, 2.21 ± 0.57 l · min^−1^), breathing frequency (32.8 ± 6.9, 38.3 ± 6.2, 39.3 ± 4.3 breathes) or V_E_ (46.7 ± 35.7, 44.0 ± 12.8, 38.1 ± 3.3 l · min^−1^) between emphysema, IPF and healthy participants, respectively.

A significant relationship was present between peak $\overset{.}{V}$ O_2_ and the $\overset{.}{V}$ O_2_ τ (*r* = −0.80; *P* = 0.000), $\overset{.}{V}$ O_2_ MRT (*r* = −0.73; *P* = 0.000), relative slow component percentage (*r* = 0.63; *P* = 0.008) and HR τ (*r* = −0.85; *P* = 0.000). Furthermore, the $\overset{.}{V}$ O_2_ τ and HR τ were significantly related (*r* = 0.81; *P* = 0.000). There was no relationship between the $\overset{.}{V}$ O_2_ τ and the [HHb]/ $\overset{.}{V}$ O_2_ ratio.

## Discussion

This is the first study to explore the influence of emphysema and IPF on the dynamic adjustments of pulmonary $\overset{.}{V}$ O_2_, HR and localized muscle fractional oxygen extraction during heavy intensity cycling exercise. We found that both patient groups had impaired exercise responses with slower $\overset{.}{V}$ O_2_, and HR kinetics relative to their healthy counterparts. Interestingly and contrary to our experimental hypothesis, the $\overset{.}{V}$ O_2_ and [HHb] response profiles in emphysema and IPF were similar, although the former demonstrated a significantly slower HR response. These findings suggest that, whilst emphysema and IPF have quite different clinical and histopathological features, exercise rehabilitation programs may wish to target the enhancement of systemic O_2_ delivery and peripheral muscle O_2_ availability to minimize the functional aerobic fitness impairments typical of people suffering from both conditions.

The slower pulmonary $\overset{.}{V}$ O_2_ kinetics in our patients with emphysema are in accord with those previously reported in COPD patients during both moderate [3, 5] and heavy intensity exercise [4]. The present study is novel in that we utilised multiple (rather than single) heavy intensity exercise transitions and recruited only those patients with clearly defined emphysema. Consequently, our resulting confidence intervals for τ in the dynamic responses of $\overset{.}{V}$ O_2_ (5 ± 2 s), HR (5 ± 3 s) and [HHb] (2 ± 1 s) are commensurate with those recommended by Fawkner and Armstrong [35], although it is pertinent to note the potential influence of our chosen interpolation techniques on these confidence intervals [36, 37]. Indeed, while three exercise transitions have been shown be associated with the lowest confidence intervals [at least compared to 1, 2, 4 or 6 transitions; 38], the applicability of such findings to the small amplitudes reported in the present study is likely to be limited [39].

The factors that determine the $\overset{.}{V}$ O_2_ response are still contentious, yet will be fundamental to the future development of effective pulmonary rehabilitation exercise programs for people with respiratory disease. Previous studies of moderate and heavy intensity exercise have suggested that oxygen delivery may represent the primary rate-limiting factor of the $\overset{.}{V}$ O_2_ kinetics in COPD [3, 4, 16]. This assertion is largely based on observations of impaired HR kinetics, which are thought to provide a good proxy of the kinetics of cardiac output and muscle blood flow [40, 41]. However, Chiappa et al. [4] have suggested that focusing on the dynamic HR may be inappropriate as they attributed the slower kinetics of cardiac output in their patients predominantly to a slower increase in stroke volume (52% slower τ) rather than heart rate (40% slower τ). Our observation that patients with emphysema had a 70% slower HR kinetic response compared to healthy controls implies a considerable limitation in central oxygen delivery following the onset of exercise. This notion is further supported by the [HHb]: $\overset{.}{V}$ O_2_ ratio which demonstrated an overshoot in both patient groups at the onset of exercise, with mean values >1.0 across the exercise transition. This is indicative of a mismatch between microvascular O_2_ distribution and muscle O_2_ utilisation in the active tissues, requiring a greater reliance on O_2_ extraction for a given relative $\overset{.}{V}$ O_2_ [26, 34]. The values reported in the present study, at least for the IPF patients, are similar to those recently reported in untrained older adults [42]. It is interesting to note the considerably lower values in the emphysema patients, although we accept this may be a methodological artefact due to the small sample size and low signal-to-noise ratio.

Recent findings in healthy older adults suggest that impairments in the dynamics of O_2_ delivery may be related to the slowing of $\overset{.}{V}$ O_2_ kinetics generally observed with ageing [43–46]. Given that COPD and IPF typically present in older adults, it is feasible that the pathological derangements associated with these diseases may engender further impairments in the dynamics of O_2_ delivery and, thus, the $\overset{.}{V}$ O_2_ kinetic response. Certainly, multiple perturbations, such as an altered autonomic balance [47] and altered mechanics and muscle fibre recruitment patterns of breathing [6, 48, 49], have been identified in people with COPD and could contribute to an O_2_ delivery limitation. Furthermore, people with COPD are known to have an increased cardiac sympathetic tone [30] and at the onset of exercise, cardiac parasympathetic withdrawal may be less pronounced compared to their healthy counterparts. Given that sympathetic activation is considerably slower at increasing heart rate than parasympathetic withdrawal [50], this autonomic imbalance could explain the slower HR response at the onset of exercise. Expiratory flow limitations can result in higher intrathoracic pressures during expiration in those with COPD which could impair right ventricular filling and left ventricular ejection [6] and hence further reduce O_2_ delivery. Indeed, simulated expiratory flow limitations in healthy adults caused significantly slower recovery $\overset{.}{V}$ O_2_ kinetics [51]. These negative cardio-circulatory consequences may additionally, or alternatively, be elicited through the significant contribution of the abdominal muscles to breathing in some patients with COPD which similarly increases expiratory pressure [48]. Finally, the increased work of breathing in those with COPD [6], especially during exercise, may divert a greater proportion of blood flow to the respiratory muscles, potentially decreasing blood flow and thus O_2_ delivery to the exercising muscles [52].

The slower dynamic $\overset{.}{V}$ O_2_ response in patients with emphysema may also be caused by factors independent of cardiac output. For example, increased sympathetic tone may result in vasoconstriction, impairing muscle blood flow and thus reducing O_2_ delivery [53]. Alternatively, it has been reported that patients with emphysema have elevated plasma levels of tumor necrosis factor-α and reactive oxygen species [54], which decrease the availability of nitric oxide (NO), an important vasodilator in muscle. However, whilst Berry et al. [55] and Kerley et al. [56] proposed that dietary nitrate supplementation might be beneficial, it has recently been demonstrated that this is ineffective in people with COPD [57].

The notion of an intramyocyte limitation is supported by the present study in which we observed a slower [HHb] response in patients with emphysema compared to published values for healthy older adults [~8–15 s;^43^, ^45^]. It is known that patients with COPD have a lower muscle oxidative capacity due to a decreased oxidative enzyme activity, reduced mitochondrial density and increased proportion of type II muscle fibers [58]. The latter have a lower oxidative but a higher glycolytic capacity and (at least in mouse muscle) an inherently slower $\overset{.}{V}$ O_2_ response relative to type I muscle fibers [59, 60]. Therefore, it could be speculated that a greater reliance on type II muscle fibers in patients with emphysema during heavy intensity exercise explains our observed slowing of the dynamic $\overset{.}{V}$ O_2_ response. Further evidence to support this notion was provided by Casaburi et al. [5] who reported a significantly faster $\overset{.}{V}$ O_2_ response in patients with COPD following exercise training despite no change in HR kinetics. Whilst the present study was not designed to investigate the mechanistic basis of the slower $\overset{.}{V}$ O_2_ kinetics in people with pulmonary disease, it seems likely to involve impairments in both oxygen delivery and myocyte function.

Dyspnea is the most common and debilitating symptom in patients with IPF in whom it is the primary impairment to quality of life [61]. It is therefore surprising that there is such a paucity of information regarding the physiological response to heavy exercise in IPF and whether the aforementioned mechanisms relating to emphysema also apply to this condition. Our findings relating to $\overset{.}{V}$ O_2_ τ suggest that patients with emphysema and IPF exhibit similar overall functional impairment, although modulatory factors may differ in the two conditions. Specifically, our patients with emphysema demonstrated an approximately 27% slower HR response than those with IPF and this might indicate a greater relative impairment of oxygen delivery in these patients although our small sample size dictates this difference should be interpreted with caution.

Contrary to expectation and the findings of Chiappa et al. [4], a negligible O_2_ slow component was observed in both patient groups. Whilst this could be due to methodological limitations related to measurement of relative exercise intensity in our patients, it is pertinent to note the exercise until volitional exhaustion in the study of Chiappa et al. [4]. Given recent advances in our understanding of the etiology of the slow component [62, 63], further work is warranted to ascertain the applicability of such findings to patients with lung disease.

Whilst we believe the present study extends our understanding of the exercise intolerance characteristic of IPF patients and provides an insight into a clearly defined emphysema patient group, it has certain limitations. Specifically, [HHb] data was not collected in the healthy control participants, arterial desaturation was not measured and the population sizes were relatively small; all these factors could potentially influence the conclusions we have drawn. Indeed, it is important that our results are considered with caution given the lower statistical power associated with the small sample size. A greater sample size may also allow questions with regard to the optimal modeling strategy for [HHb] data to be investigated, with concerns raised that the present method may not optimally describe the initial transient response. Moreover, the low response amplitude and subsequent signal-to-noise ratio requires caution in the interpretation of the present findings, especially with regards to potential discrepancies in relative exercise intensity which may have influenced the presence of a $\overset{.}{V}$ O_2_ slow component. It is also pertinent to note the age of the current participants; underlying age-related declines in the dynamic exercise response limit the attribution of the observed impairments to solely COPD. Furthermore, the present study used heart rate kinetics as a proxy for oxygen delivery and we did not formally assess dyspnea during exercise. Finally, to limit the burden on our patients who had relatively severe lung disease, a supra-maximal validation bout for peak $\overset{.}{V}$ O_2_ was not performed. Despite these limitations, we believe this pilot study provides important information, which could inform future research into functional limitations of people with IPF and emphysema.

## Conclusions

In conclusion, the pathological conditions of emphysema and IPF both impair the normal dynamic respiratory and cardiac responses following the onset of exercise. Specifically, they are characterised by significantly slower $\overset{.}{V}$ O_2_, HR and [HHb] kinetics relative to healthy controls, suggesting that both oxygen delivery and metabolic inertia limit the O_2_ response in these patients. Our findings may help in the development of future therapeutic strategies designed to improve functional aerobic fitness in people suffering from emphysema and IPF. Specifically, regimes that target enhanced systemic O_2_ delivery and peripheral muscle O_2_ availability may be particularly effective.

## Abbreviations

$\overset{.}{V}$ O_2_: Rate of oxygen uptake
[HHb]: Deoxyhemoglobin
Amp: Amplitude
BF: Body fat
COPD: Chronic obstructive pulmonary disease
DLco: Carbon monoxide transfer factor
FEV_1_: Forced expiratory volume in one second
FVC: Forced vital capacity
GET: Gas exchange threshold
HR: Heart rate
IPF: Idiopathic pulmonary fibrosis
Kco: Carbon monoxide transfer coefficient
MRT: Mean response time
NIRS: Near infrared spectroscopy
NO: Nitric oxide
O_2_: Oxygen
TD: Time delay
VCO_2_: Rate of carbon dioxide production
V_E_: Minute ventilation
V_T_: Tidal volume
τ: Time constant
